# 82. Assessment of Clinical Outcomes and Antibiotic Prescribing Patterns Following Implementation of the GenMark ePlex^®^ Blood Culture Identification Panel for Gram-positive Bloodstream Infections

**DOI:** 10.1093/ofid/ofab466.284

**Published:** 2021-12-04

**Authors:** Megan Wein, Shawn Binkley, Vasilios Athans, Stephen Saw, Tiffany Lee, Sonal Patel, Keith W Hamilton, Amanda Binkley, Kathleen Degnan, Laurel Glaser, Lauren Dutcher, Naasha J Talati, Melissa Richard-Greenblatt

**Affiliations:** 1 Hospital of the University of Pennsylvania, Philadelphia, PA; 2 Penn Presbyterian Medical Center, Philadelphia, PA; 3 University of Pennsylvania, Philadelphia, PA

## Abstract

**Background:**

Rapid diagnostic testing (RDT) of bloodstream pathogens provides key information sooner than conventional identification and susceptibility testing. The GenMark ePlex® blood culture identification gram-positive (BCID-GP) panel is a molecular-based multiplex platform, with 20 Gram-positive target pathogens and 4 bacterial resistance genes that can be detected within 1.5 hours of blood culture positivity. Published studies have evaluated the accuracy of the ePlex® BCID-GP panel compared to traditional identification methods; however, studies evaluating the impact of this panel on clinical outcomes and prescribing patterns are lacking.

**Methods:**

This multi-center, quasi-experimental study evaluated clinical outcomes and prescribing patterns before (December 2018 – June 2019) and after (August 2019 – January 2020) implementation of the ePlex® BCID-GP panel in June 2019. Hospitalized, adult patients with growth of *Enterococcus faecalis, Enterococcus faecium,* or *Staphylococcus aureus* from blood cultures were included. The primary endpoint was time to targeted antibiotic therapy, defined as time from positive Gram-stain to antibiotic adjustment for the infecting pathogen.

**Results:**

A total of 200 patients, 100 in each group, were included. Time to targeted therapy was 47.9 hours in the pre-group versus 24.8 hours in the post-group (p< 0.0001). Time from Gram-stain to organism identification was 23.03 hours (pre) versus 2.56 hours (post), p< 0.0001. There was no statistically significant difference in time from Gram-stain to susceptibility results, hospital length of stay (LOS), or all-cause 30-day mortality.

**Conclusion:**

Implementation of the GenMark ePlex® BCID-GP panel reduced time to targeted antibiotic therapy by nearly 24 hours. Clinical outcomes including hospital LOS and all-cause 30-day mortality did not show a statistical difference, although analysis of a larger sample size is necessary to appropriately assess these outcomes. This study represents the effect of RDT implementation alone, in the absence of stewardship intervention, on antibiotic prescribing patterns. These findings will inform the design of a dedicated RDT antimicrobial stewardship intervention at our institution, while also being generalizable to other institutions with RDT capabilities.

**Disclosures:**

**All Authors**: No reported disclosures

